# The hidden costs of Dutch dietary choices: quantifying the health and environmental costs attributable to suboptimal diets

**DOI:** 10.3389/fnut.2026.1759095

**Published:** 2026-03-23

**Authors:** L. Jacky Florencio, Jeljer Hoekstra, Elisabeth H. M. Temme, Reina E. Vellinga, G. Ardine de Wit

**Affiliations:** 1 National Institute for Public Health and the Environment, Bilthoven, Netherlands; 2 Department of Health Sciences, Faculty of Science, Vrije Universiteit Amsterdam, Amsterdam, Netherlands

**Keywords:** dietary guidelines, externalities, health effects of diets, hidden costs, impact assessment, sustainable food consumption, true cost accounting

## Abstract

**Background:**

National dietary guidelines can help in promoting healthier and more sustainable dietary patterns. Although the potential benefits of adherence are well documented, observed adherence in the Netherlands remains suboptimal.

**Objective:**

This study estimated the annual hidden health and environmental costs associated with suboptimal dietary patterns, defined here as low adherence to the Dutch dietary guidelines.

**Methods:**

Adherence to the Dutch dietary guidelines was assessed using the Dutch Healthy Diet index 2015 (DHD15). Individuals in the lowest (Q1) and highest (Q5) quintiles of adherence were compared based on food consumption data from the Dutch National Food Consumption Survey (DNFCS) 2019–2021 among adults aged 20–79 years. Hidden health costs were estimated as productivity losses using the Human Capital Approach (HCA) for diet-related non-communicable diseases (NCDs), along with the economic valuation of disability-adjusted life years (DALYs). Environmental costs were calculated across six indicators, including greenhouse gas (GHG) emissions, terrestrial acidification, freshwater eutrophication, marine eutrophication, land use, and blue water consumption, and were monetised using environmental shadow prices.

**Results:**

Low adherence to dietary guidelines is associated with EUR 410 million in productivity losses, with DALY-related costs ranging from EUR 910 million to 1.8 billion. Low adherence imposes EUR 3.0 billion more in annual environmental costs compared to high adherence.

**Discussion:**

These results reinforce calls for policies and interventions that promote healthier eating patterns as a cost-effective means of reducing the hidden costs embedded in current dietary habits and transforming the agrifood system toward health and sustainability goals.

## 1 Introduction

Dietary intake plays an important role in shaping long-term population health (1). Inadequate or imbalanced diets are a major contributor to the global burden of non-communicable diseases (NCDs), including cardiovascular diseases, cancers, and type 2 diabetes (2). These diseases are the leading causes of mortality and morbidity, especially among the working-age population (3). Beyond their impact on public health, these diseases lead to increased healthcare costs (4), placing a higher financial burden on a country’s budget and welfare (5). Indirectly, premature death and illness reduce the availability and productivity of labour (6). This impact is rooted in the principle that healthier individuals possess greater physical and mental capacity which enables them to generate more output per hour worked (7) compared to individuals with suboptimal health. A broader perspective on the economic burden is captured through the concept of hidden costs, which is defined as costs to individuals or society that are not reflected in market prices, which often arise from external costs or failures in markets or policies (8). The Food and Agriculture Organization of the United Nations (FAO) estimates the global hidden costs of food systems at 11.6 trillion dollars at 2020 purchasing power parity (PPP) (8), with unhealthy dietary patterns accounting for 70 percent of these costs. In parallel to the health impacts, suboptimal diets are closely linked to environmental sustainability (9). Dietary patterns common in Western countries, which are typically high in red and processed meats and low in fruits, vegetables, and legumes, are both unsustainable and unhealthy (10). Such patterns are not in line with national dietary guidelines, and are associated with higher greenhouse gas (GHG) emissions and land use (11). Since these environmental pressures are not reflected in market prices, consumers receive distorted price signals that underestimate the environmental impact of their food choices (12) and contribute to unsustainable food consumption.

In this context, national dietary guidelines can play an important role in promoting healthier and sustainable dietary patterns and also reduce the hidden health and environmental costs (13). In the Netherlands, the 2015 dietary guidelines promote a transition toward more plant-based and less animal-based dietary patterns, recommending daily intake of vegetables, fruit, wholegrains, and nuts, alongside weekly intake of legumes, and fish. They also advise limiting the intake of red and processed meat, sugar-sweetened beverages (SSBs), alcohol, and salt, and replacing refined grains, animal fats, and unfiltered coffee with healthier alternatives (14). While the national guidelines primarily aim to promote health outcomes, sustainability considerations are considered to a limited extent (15). Although the potential health and environmental benefits are well established (11, 16–18), adherence to the Dutch guidelines remains suboptimal, with fewer than half of the Dutch population meeting the recommended dietary intakes (19). In 2022, unhealthy dietary patterns were responsible for almost 149,000 disability-adjusted life years (DALYs), representing 2.8 percent of the total Dutch national disease burden (20). A recent modelling study estimated that adhering to the Dutch dietary guidelines, including, for instance, the elimination of processed meat consumption and meeting recommended fruit intake levels, could reduce the burden of NCDs by 2050, with projected reductions of 19–24 percent in coronary heart disease (CHD), 19–25 percent in type 2 diabetes, and 18–19 percent in stroke incidence (17). Additionally, higher adherence to the Dutch national guidelines has been associated with a reduction of GHG emissions, although it would be accompanied by an increase in blue water use (21).

While the health and environmental consequences of suboptimal diets are commonly recognised, there is still limited literature on the full societal costs associated with unhealthy dietary patterns, particularly in relation to the hidden costs. Cost-of-illness (COI) studies conducted in the Netherlands typically focus on direct health and social expenditures (20, 22). However, broader economic implications, such as productivity losses, are less frequently addressed, despite their potential to exceed healthcare-related costs (23) and reduce overall societal welfare. Additionally, health costs assessments frequently examine individual dietary risk factors in isolation rather than considering the effects of overall dietary patterns (8, 20). Although the environmental impacts of diets are increasingly reported in terms of resource use and emissions, their conversion into monetary terms remains limited. This constrains the integration of environmental costs into broader economic evaluations and policy discussions. The aim of this study was to estimate the hidden costs associated with dietary patterns that deviate from the Dutch dietary guidelines. We make a first attempt to evaluate the costs at the level of overall dietary patterns rather than examining individual dietary risk factors. Specifically, the hidden health and environmental costs associated with suboptimal dietary patterns are assessed by comparing individuals with the lowest adherence to the Dutch dietary guidelines (= poorest dietary habits) to those with the highest adherence (= best dietary habits). For the health hidden costs, the productivity losses related to diet-related NCDs were estimated. A complementary analysis was conducted by assigning an economic value to DALYs, which incorporate aspects of health that are not directly captured in productivity measures. For the environmental hidden costs, six environmental indicators were considered, namely: GHG emissions, terrestrial acidification, freshwater eutrophication, marine eutrophication, land use, and blue water consumption.

## 2 Materials and methods

### 2.1 General approach

This section provides a description of the general approach. The following sections provide the full definitions, data sources and the analysis methods. An overview of the data sources used is provided in Supplementary Table 1.

**Figure 1:**
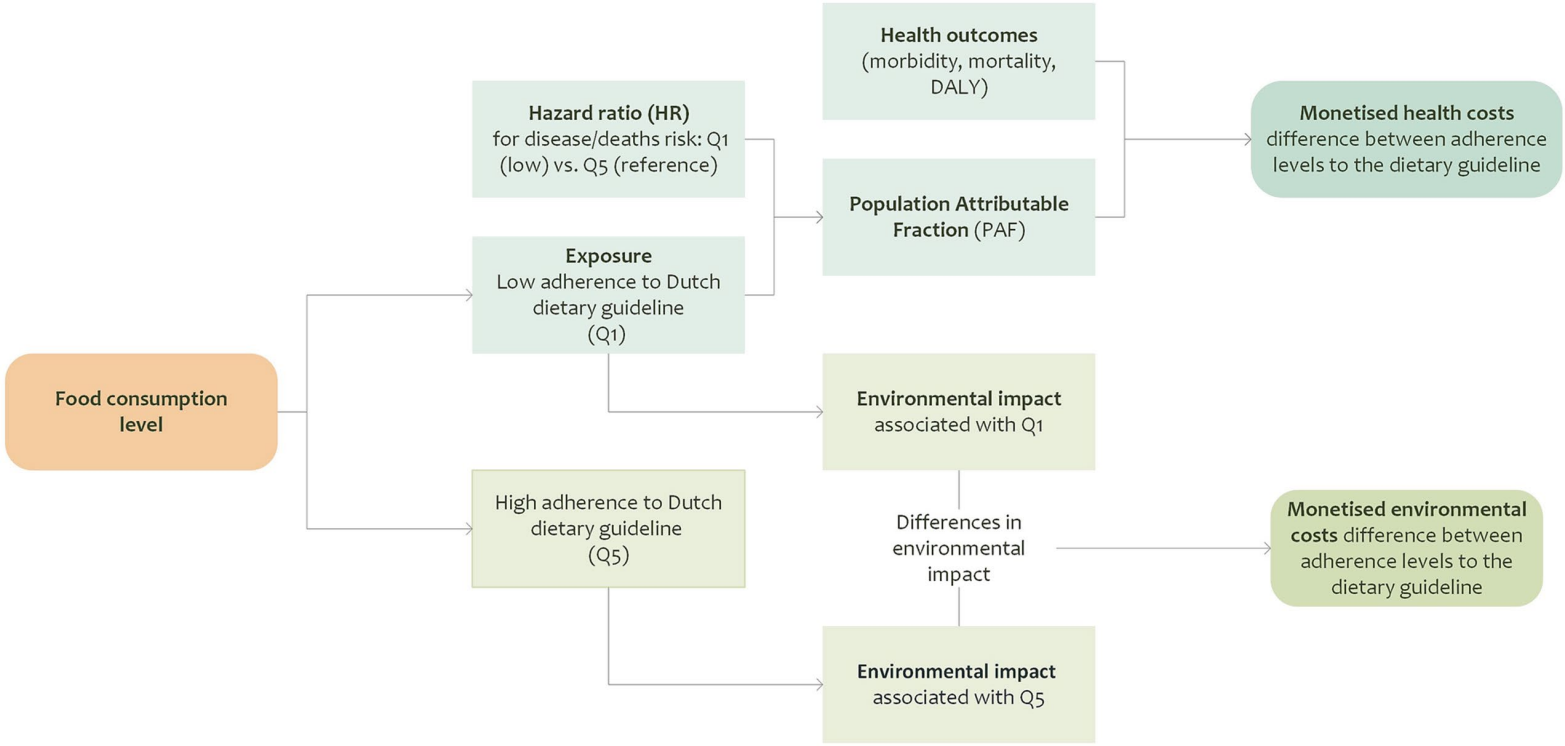
Methodological framework for estimating the monetised health and environmental costs associated with different levels of adherence to the Dutch dietary guidelines.

This study estimates the health-related and environmental costs, referred to as hidden costs in this paper, associated with suboptimal dietary patterns. The analysis contrasts individuals with the lowest adherence to the Dutch dietary guidelines (quintile 1, Q1) to those with the highest adherence (quintile 5, Q5) based on a dietary adherence score, the Dutch Healthy Diet Index 2015 (DHD15), which was calculated from self-reported food consumption data. The specific contrast between Q1 and Q5 was chosen since a previous study demonstrated statistically significant differences in disease and all-mortality risks between these groups (16). The methodological framework includes two main components, as shown in Figure 1.

The first component involves the assessment of health-related hidden costs, for which two approaches were used to capture both market-based and non-market costs. For the first approach, market prices were used to estimate the productivity losses resulting from suboptimal adherence to the Dutch dietary guidelines. Hazard ratios (HR) for diet-related diseases and deaths were used to calculate the Population Attributable Fraction (PAF), which was used to assess the proportion of disease burden associated with low dietary adherence. These attributable cases formed the basis for estimating productivity losses due to morbidity and mortality. For comparison purposes, the analysis also included healthcare costs^1^ associated with low adherence, using the same methodological framework. For the second approach, shadow prices were used to estimate the indirect costs associated with health outcomes which are not directly observable in market transactions. As in the first approach, the morbidity PAFs were used to estimate the proportion of disease burden associated with low adherence. This attributable burden, expressed in DALYs, was then monetised to capture the broader economic impact of poor dietary adherence. For the environmental component, the environmental impacts associated with observed food consumption patterns in Q1 and Q5 were estimated. The differences in these impacts were monetised using Dutch environmental shadow prices to quantify the hidden costs. The calculations presented in this study were conducted for the reference year 2023.

### 2.2 Food consumption and assessment of adherence to the dietary guidelines

Food consumption data were derived from the Dutch National Food Consumption Survey (DNFCS) 2019–2021. A full explanation of the DNFCS study is described elsewhere (19). In short, the DNCFS study drew participants from a Kantar consumer panel, ensuring representation across age, gender, education, region, and urbanisation levels. Participants completed a digital questionnaire and were interviewed by trained dietitians using the GloboDiet system. For this study, consumption data of all participants aged 20 to 79 years were included in the analysis (*n* = 1715). Adherence to the Dutch dietary guidelines was assessed using the DHD15 (24). This index includes fifteen components: vegetables, fruits, wholegrain products, legumes, nuts, dairy, fish, tea, fats and oils, coffee, red meat, processed meat, sweetened beverages and fruit juices, alcohol, and sodium. Each component reflects a specific guideline and is scored from 0, indicating no adherence, to 10, indicating full adherence, implying that the maximum DHD15 score is 150 (25). In this study, a maximum total score of 140 points was applied since specific information on coffee consumption was not available in the DNFCS.

### 2.3 Disease burden attributable to suboptimal diets

The morbidity and mortality burden attributable to suboptimal adherence to the Dutch dietary guidelines was calculated using PAFs. PAFs estimate the proportion of disease cases or deaths that could theoretically be averted if exposure to a particular risk factor was modified from its current level to a defined counterfactual scenario (26). Three chronic diseases related to dietary patterns were considered, namely: stroke, Chronic Obstructive Pulmonary Disease (COPD), and colorectal cancer. While more diseases may be related to food intake patterns, the inclusion of these three diseases was based on the inverse and statistically significant results observed in a previous study (16). The choice was also guided by the availability of HRs related to the adherence to the Dutch dietary guidelines. As a sensitivity analysis, type 2 diabetes mellitus (T2DM) was additionally in our estimations (included in Supplementary materials). Previous studies have shown that better adherence to the Dutch dietary guidelines is associated with lower all-cause mortality risks (11, 16). For consistency, the results of the study by Voortman et al. (16) were applied in this study. It was assumed that all-cause mortality reflected deaths from NCDs with well-established dietary links.

The NCDs were selected based on Duan et al. (2025) (17) and included CHD, stroke, T2DM, colorectal cancer and lung cancer. Hence, this implies a broader range of diseases is included for all-cause mortality than for direct relationships between dietary patterns and disease. Age-and sex-specific prevalence and deaths in 2023 were obtained from VZinfo.nl (27–29) and the Netherlands Cancer Registry (NCR) (30). To calculate the PAFs, information on relative risks (RR) is required. Since RRs were not available, HRs were used; these estimate the relative rate of events over time between differently exposed groups and can be interpreted similarly to RRs (31). The HRs used in this study were retrieved from Voortman et al. (16), who assessed the association between adherence to the Dutch dietary guidelines and NCDs incidence and mortality. In their analysis, HRs were reported across quintiles of dietary adherence, with the lowest quintile serving as the reference group (HR = 1). For this study, and to allow interpretation based on the lowest exposure group, the HRs were recalculated using the highest adherence group (e.g., Q5, HR = 0.71) as reference category by inverting the reported values (e.g., Q1: 1/0.71 = 1.41). Since age- and sex-specific HRs were not available, it was assumed that the HRs per adherence quintile were constant across age and sex groups. Latency between exposure and outcome was not modelled in the estimations, as it was assumed that the HRs reflect the accumulated impact of dietary patterns over time.

The morbidity and mortality PAFs were estimated using the following formula:


$$\mathrm{PA}F_{i}=\frac{P_{i}\cdot (HR_{i}-1)}{P_{i}\cdot (HR_{i}-1)+1}$$

Where $P_{i}$ represents the proportion of the population in adherence category i [in this case, the lowest adherence quintile (Q1)], and $HR_{i}$ is the HR of morbidity or mortality for that category compared to the highest adherence group (Q5). To account for variation in dietary adherence across the population, $P_{i}$ was calculated for each sex and age group. This was done by dividing the number of individuals in Q1 within each subgroup by the total number of individuals in that subgroup. This approach allows the estimation of the exposure-specific PAFs that reflect actual differences in dietary risk across demographic groups, rather than assuming a uniform exposure level (e.g., 20 percent) across the entire population. These group-specific PAFs were then applied to the observed number of disease cases and deaths in the Dutch population for each sex and age group.

### 2.4 Productivity losses due to morbidity and mortality

To estimate the costs associated with health impacts of low adherence to the dietary guidelines, productivity loss was calculated using the Human Capital Approach (HCA). The productivity losses due to morbidity were estimated using lost production days, daily labour cost per day, and the estimated absolute attributable disease burden. For this study, 30-day productivity loss was applied for an incident case of stroke, 40.6 days for COPD, and 47.6 days for colorectal cancer based on the results from the epidemiological study NEMESIS-2 (Netherlands Mental Health Survey and Incidence Study-2) (32), which examined absenteeism due to do both mental and somatic conditions among employed individuals. The labour cost per day in 2022 was retrieved from the National Health Care Institute (33) and was adjusted for inflation over the years (34). A 36-h workweek (35) and a 7.2-h workday was assumed, resulting in an estimated labour cost of EUR 298.16 per day. Productivity losses from premature deaths were estimated using the estimated attributable deaths and discounted economic loss per death. The discounted economic loss per death was based on the remaining working years at time of death, employment rates, and annual production value per employee. The remaining working years were estimated up to the Dutch pension age of 67 and adjusted for age-specific economic activity and employment rates with data retrieved from Statistics Netherlands (36). Age-and sex-specific annual production losses were based on inflation-adjusted hourly labour costs and considering an average annual working hours of 1,822 (35). Projected earnings were discounted at 3 percent annually, in line with the Dutch guidelines for economic evaluations in healthcare (33). Data for the comparison analysis considering healthcare costs (e.g., hospital care, primary care, medicine and medical aids, ambulance care, other healthcare providers, public care and prevention, elderly care, and healthcare management) were retrieved from the 2019 Dutch COI study (22), which specifies these healthcare costs for different chronic conditions. A sensitivity analysis using the friction cost approach, which estimates productivity losses only for the period needed to replace the worker who is absent due to illness (37) was carried out and is available in the Supplementary materials.

### 2.5 Monetisation of disability-adjusted life-years

In this study, the DALYs were monetised using Quality-Adjusted Life Years (QALYs) valuation factors. QALYs combine the length of life and the quality of life into a single metric, serving as a standardised measure of health-related utility or benefit (38). QALYs and DALYs serve as complementary metrics in the assessment of population health. QALYs quantify the years lived in full health, whereas DALYs represent the years of healthy life lost due to morbidity and premature mortality. Theoretical distinctions between the two have been described in previous studies (39–41); however, discrepancies are typically modest and rarely affect conclusions against common cost-effectiveness thresholds (42). Conceptually, the two can be regarded as functional opposites (43). Age-and sex-specific DALYs in 2022 were obtained from VZinfo.nl (44). A monetary value was assigned to each QALY, considering values of EUR 50,000 to 100,000 as recommended in the SEO Amsterdam Economics guidelines for social cost–benefit analyses in the Netherlands (SCBAs) (45). These values do not represent budgetary costs or tariffs but reflect observed societal willingness-to-pay (WTP).

### 2.6 Environmental impact associated with different dietary patterns

The environmental impact of different dietary adherence levels (Q1 and Q5) was calculated for six indicators: GHG emissions (kg CO₂-eq.), terrestrial acidification (kg SO₂-eq.), freshwater eutrophication (kg P-eq.), marine eutrophication (kg N-eq.), land use (m^2^a crop eq.), and blue water consumption (m^3^). Environmental impact data from the Dutch life cycle assessment (LCA) Food Database version 2024 (46, 47), was used. A full explanation of the database is given elsewhere (48). The database includes approximately 400 foods that represent a major share of the daily Dutch food consumption. The LCAs were conducted using an attributional approach and a hierarchical perspective, in alignment with the ISO-14040 and 14,044 principles, and where applicable, aligned with the Product Environmental Footprint. A functional unit of 1 kg of prepared food, as consumed by the Dutch consumer and sold through a Dutch supermarket, was used. The system boundaries of the LCAs encompassed all stages of the product’s life cycle - from cradle to plate - including primary production, (post-harvest) processing, packaging, storage and distribution, retail, and consumer preparation, consumption and end-of-life/waste processing. Economic allocation was applied when products led to by-products. Environmental impact data were previously linked to foods consumed in the DNFCS 2019–2021 (25). In case where no data was available, foods were linked to similar food items based on the type of food, cultivation, or origin. More detailed information on the LCA data and methodologies can be found elsewhere (21, 49). To monetise the environment impacts associated with the dietary intake among individuals with low and high adherence to the Dutch dietary guideline, the Environmental Prices Handbook 2024: EU 27 version by CE Delft was used (50), which presents the monetised damage costs associated with the release of an extra kilogram of a substance into the environment. The 2021 upper, central, and lower environmental prices were adjusted for inflation for the estimation. The environmental price of CO₂ emission was adjusted by applying an autonomous annual price increase of 3.5%, as proposed in the handbook. The price of the remaining five indicators was corrected for income based on changes in actual disposable income over the years (51). The resulting environmental costs were estimated at the population level, using population data from Statistics Netherlands (52), and on an annual basis.

### 2.7 Statistical analysis

Daily means over two consumption days were calculated for each FFQ participant to estimate food consumption, DHD15 scores adjusted for sex, and the environmental impact. The daily environmental impact of food consumption for all six indicators was scaled to reflect the annual environmental burden. The total costs at the population level were estimated by taking age- and sex- specific population distributions in Q1 and Q5 into account. Results are presented as mean (standard deviation (SD)) and 95% Confidence Intervals (CI), where applicable.

## 3 Results

### 3.1 Adherence to the Dutch dietary guidelines and the associated environmental impact

In the lowest quintile (Q1), DHD15 scores ranged from 27.9 to 65.1, whereas the highest quintile (Q5) scores ranged from 81.2 to 125.0 (see Table 1). On average, Dutch individuals aged 20–79 years had a mean DHD15 score of 72.8 (95% CI: 72.1–73.5). Individuals in the lowest adherence group had a mean score of 52.8 (95% CI: 52.0–53.6), while those with the highest adherence had a mean score of 93.1 (95% CI: 92.3–93.9).

**Table 1:** Baseline characteristics for individuals with the lowest (Q1) and highest (Q5) adherence to the Dutch dietary guidelines.

	Total *N* = 1715	Q1 *N* = 343	Q5 *N* = 343
DHD15 score			
Range^1^		27.9–65.1	81.2–125.0
Mean (95%-CI)	72.8 (72.1–73.5)	52.8 (52.0–53.6)	93.1 (92.3–93.9)
Sex			
Females	844 (49.2%)	169 (49.1%)	168 (49.1%)
Males	871 (50.8%)	175 (50.9%)	174 (50.9%)
Age group			
20–24	52 (3.0%)	16 (4.7%)	4 (1.2%)
25–29	56 (3.3%)	9 (2.6%)	10 (2.9%)
30–34	82 (4.8%)	19 (5.5%)	14 (4.1%)
35–39	111 (6.5%)	31 (9.0%)	21 (6.1%)
40–44	107 (6.2%)	25 (7.3%)	17 (5.0%)
45–49	133 (7.8%)	27 (7.8%)	24 (7.0%)
50–54	189 (11.0%)	40 (11.6%)	36 (10.5%)
55–59	182 (10.6%)	45 (13.1%)	28 (8.2%)
60–64	196 (11.4%)	44 (12.8%)	30 (8.8%)
65–69	276 (16.1%)	49 (14.2%)	66 (19.3%)
70–74	213 (12.4%)	25 (7.3%)	62 (18.1%)
75–79	118 (6.9%)	14 (4.1%)	30 (8.8%)

Values are given in mean (95% CI) and percentages; Q, Quintile; DHD15, Dutch Healthy Diet Index 2015; ^1^Range of DHD15 score; Values are given as number of participants (N) with percentages in parentheses.

Table 2 shows that the environmental impacts differed between adherence levels. Individuals in the highest adherence group had lower environmental impacts across most indicators in comparison to those in the lowest adherence group. Mean GHG emission were 3.7 kg CO₂-eq./day in Q5 versus 4.7 kg CO₂-eq./day in Q1, and land use was 3.2 vs. 3.7 m^2^ year/day, respectively. Freshwater eutrophication (1.4 vs. 1.5 kg P-eq./day), marine eutrophication (3.9 vs. 5.3 kg N-eq./day), and terrestrial acidification (12.8 vs. 17.0 kg SO₂-eq./day) were also lower in Q5. However, blue water consumption was higher in Q5, with a mean of 0.12 m^3^/day compared to 0.08 m^3^/day in Q1.

**Table 2:** Daily average environmental impact of dietary intake across the total population and by adherence to the Dutch dietary guidelines (Q1 and Q5).

	Total		Q1		Q5	
	Mean (SD)	95% CI	Mean (SD)	95% CI	Mean (SD)	95% CI
GHG emission (kg CO_2_-eq./d)	4.1 (1.4)	(4.00–4.12)	4.7 (1.7)	(4.5–4.8)	3.7 (1.1)	(3.6–3.8)
Land use (m^2^*year/d)	3.3 (1.1)	(3.28–3.38)	3.7 (1.3)	(3.6–3.9)	3.2 (0.9)	(3.1–3.3)
Freshwater eutrophication (kg P-eq./d)	1.4 (0.6)	(1.41–1.47)	1.5 (0.6)	(1.5–1.6)	1.4 (0.6)	(1.4–1.5)
Marine water eutrophication (kg N-eq./d)	4.5 (1.8)	(4.37–4.55)	5.3 (2.2)	(5.1–5.5)	3.9 (1.4)	(3.8–4.1)
Terrestrial acidification (kg SO2-eq./d)	14.5 (5.1)	(14.26–14.73)	17.0 (6.0)	(16.4–17.6)	12.8 (3.7)	(12.4–13.2)
Blue water consumption (m^3^/d)	0.10 (0.06)	(0.09–0.10)	0.08 (0.04)	(0.08–0.09)	0.12 (0.07)	(0.11–0.13)

Values are given in mean (SD) (95% Confidence Intervals); Q, Quintile.

### 3.2 Monetised impacts

#### 3.2.1 Productivity losses

A total of 51,079 cases of the selected chronic diseases and 460 deaths were attributable to low adherence to the Dutch dietary guidelines (see Table 3). The total productivity losses due to low adherence to the Dutch dietary guidelines amounted to EUR 410 million, with EUR 318 million (78 percent) attributable to morbidity. COPD was the main contributor to morbidity-related productivity losses, accounting for EUR 196 million, followed by stroke (EUR 106 million). In terms of mortality-related losses, lung cancer was the leading condition, resulting in EUR 34 million in lost productivity, followed by CHD (EUR 19 million), colorectal cancer (EUR 19 million), and stroke (EUR 14 million).

**Table 3:** Attributable cases and productivity losses associated with low adherence compared to high adherence to the Dutch dietary guidelines, by sex, disease, morbidity, and mortality.

	Males		Females		Total	
	Number of cases attributable	Productivity costs (in million EUR)	Number of cases attributable	Productivity costs (in million EUR)	Number of cases attributable	Productivity costs (in million EUR)
Morbidity^1^						
Colorectal cancer	1,357	9	1,139	7	2,496	16
COPD	13,571	101	13,653	95	27,224	196
Stroke	11,788	60	9,570	46	21,359	106
					51,079	318
Mortality^2^						
CHD	70	16	24	3	94	19
Colorectal cancer	40	11	28	8	68	19
Lung cancer	101	20	90	14	191	34
Stroke	44	9	32	5	76	14
T2DM	22	5	9	1	31	6
					460	92
Total						410

CHD, Coronary Heart Disease; COPD, Chronic Obstructive Pulmonary Disease; T2DM, type 2 diabetes mellitus; ^1^Diseases were selected based on statistically significant associations observed for higher adherence (Q5) to the Dutch dietary guidelines in Voortman et al. (16); ^2^Inclusion of diseases are based on the study of Duan et al. (17).

#### 3.2.2 DALY costs

Table 4 shows the attributable DALYs, and the corresponding value associated with low adherence to the Dutch dietary guideline. In total, an estimated 18,197 DALYs were attributable to suboptimal dietary intake, corresponding to a monetised value ranging from EUR 910 million to EUR 1819 million. COPD accounted for the highest burden, with 9,478 DALYs and a value ranging from EUR 474–948 million, followed by stroke (4,887 DALYs; EUR 244–488 million) and colorectal cancer (3,832 DALYs; EUR 192–383 million).

**Table 4:** Attributable disability-adjusted life years (DALYs) and value of life associated with low adherence compared to high adherence to the Dutch dietary guideline, by sex and disease.

	Males		Females		Total	
	Attributable DALY	Value^1^ (in million EUR)	Attributable DALY	Value (in million EUR)	Attributable DALY	Value (in million EUR)
Colorectal cancer	2,158	108–216	1,674	84–167	3,832	192–383
COPD	4,416	221–442	5,062	253–506	9,478	474–948
Stroke	2,622	131–262	2,265	113–226	4,887	244–488
					18,197	910–1,819

COPD, Chronic Obstructive Pulmonary Disease; ^1^Values are given as a range based on a value of EUR 50,000 to 100,000 per Quality-Adjusted Life Years (QALYs).

#### 3.2.3 Environmental costs

As shown in Table 5, daily diets of individuals in the lowest dietary adherence group had higher environmental impacts compared to those in the highest adherence group. Total environmental costs were EUR 8547 (4431–16704) million in Q1 and EUR 5570 (2894–10920) million in Q5. Across most indicators the environmental costs were lower in Q5 compared to Q1, with differences of 31% for GHG emissions, 28% for land use, 36% for marine eutrophication, 35% for terrestrial acidification and 20% for freshwater eutrophication. In contrast, the costs associated with blue water consumption was 23%% higher in Q5 than in Q1.

**Table 5:** Annual environmental costs (in million EUR) across the total population and by adherence to the Dutch dietary guidelines (Q1 and Q5).

	Total		Q1		Q5	
	Central	Lower-Upper	Central	Lower-Upper	Central	Lower-Upper
GHG emission	27	(10–33)	32	(12–39)	22	(8–27)
Land use	16	(11–21)	18	(13–25)	13	(9–18)
Freshwater eutrophication	257	(174–730)	285	(193–809)	229	(155–650)
Marine water eutrophication	3,074	(1,628–6,235)	3,750	(1,986–7,604)	2,399	(1,270–4,865)
Terrestrial acidification	3,682	(1,839–6,789)	4,460	(2,227–8,223)	2,905	(1,451–5,356)
Blue water	1.9	(0–4.0)	1.7	(0–3.5)	2.1	(0–4.4)
	7,058	(3,662–13,812)	8,547	(4,431–16,704)	5,570	(2,894–10,920)

Values are given in mean; Q, Quintile.

## 4 Discussion

Dietary choices not only influence individual health but also broader economic health and environmental burden. Despite clear recommendations in the Dutch dietary guidelines and observed trends toward healthier eating among the population (19), overall adherence remains low and varies across population groups. This study assessed the hidden health and environmental costs associated with dietary patterns that vary in their adherence to the Dutch dietary guidelines, specifically comparing individuals with the poorest adherence to those with the highest adherence. In the discussion we address (1) the role of dietary of guidelines in reducing hidden costs, (2) considerations for deriving dietary guidelines with broader societal outcomes in mind, and (3) the limitations of this study.

### 4.1 Can dietary guidelines help reduce hidden costs?

Our results suggest that if the Dutch adult population with low dietary guidelines adherence adopted the dietary patterns observed in the highest adherence group, the combined societal cost savings would include EUR 410 million in avoided productivity losses. This is two times higher than the estimated savings in healthcare costs, which total EUR 201 million (see Supplementary Table 2). This highlights that these hidden costs, which occur since mortality and morbidity negatively affect labour productivity, can have a strong economic impact. In 2022, healthcare costs attributable to unhealthy diets in the Netherlands were estimated at 1583 million, based on 11 individual dietary risk factors and 7 diet-related conditions (53). While our results are not directly comparable due to methodological differences, an indicative comparison suggests that the avoided productivity loss among the low adherence group alone represents 26 percent of these costs. Our results are in line with previous studies that show that dietary shifts more in line with dietary guidelines not only decrease mortality risks, lower disease prevalence, reduce years of life lost and direct healthcare costs, but also mitigate indirect costs, such as those related to productivity (11, 17, 26, 54–56). When considering the DALYs, a total of 18,197 DALYs were attributable to low adherence to the Dutch dietary guidelines. These results are lower than the estimates reported in the Dutch Public Health Status and Foresight Report 2024, which attributed 148,205 DALYs to unhealthy diets in the Dutch population in 2022, representing 2.8 percent of the total disease burden (20). These differences can be explained by methodological variations and the limited scope of this study, which for instance compares only the 20 percent lowest and highest adherence groups of the Dutch dietary guideline and includes only a small selection of diseases. The corresponding value of DALY’s lost ranged from EUR 910 million to EUR 1.8 billion. A similar trend was observed by Springmann et al. (26), whose results showed that the value-of-statistical life (VSL) economic benefits associated with diets with less animal-sourced foods were higher than direct healthcare costs and productivity losses. The monetised DALYs represent a broader valuation concept than productivity losses, resulting in higher estimated values. They are derived from WTP approaches such as the Value of a Statistical Life (VSL), which is based for instance from observed wage premiums for high risk jobs, or stated-preference surveys in which individuals indicate what they are willing to pay for additional health or living longer (45). In the Dutch context, with a projected fast growth of healthcare expenditure (57), these findings are particularly salient. Moreover, the true societal burden may be underestimated, as productivity impacts are not accounted for (58), despite their implications for purchasing power and overall societal welfare (13). This highlights the importance that addressing dietary risks is essential not only for managing health burdens but also for alleviating future economic pressures.

Beyond health outcomes, various studies have focused on the impact of moving towards more healthier diets or improved dietary adherence and their potential to reduce environmental pressures (11, 26, 59–61). Sustainable dietary shifts have also been associated with, e.g., lower diet related GHG emissions, land use, and water consumption (62, 63). In the Netherlands, previous modelling studies have shown that higher adherence to the Dutch dietary guidelines could moderately reduce GHG emissions and land use (11). Another study demonstrated that a 75 percent reduction in meat consumption could reduce GHG emissions by 22–24 percent (64). Similarly, Vellinga et al. (25) showed that optimised diets aiming to increase the adherence to the national dietary guidelines and lower GHG emissions, can reduce GHG emissions by 19–24 percent, without increasing diet costs for all socio-economic subgroups. The current study builds on this evidence by monetizing these environmental impacts and showing that low adherence to the Dutch dietary guidelines imposes high environmental costs. The observed EUR 3.0 billion difference in environmental costs between Q1 and Q5 underscores that improving dietary adherence can reduce hidden costs by 35 percent. While most environmental impacts are lower with higher adherence to the Dutch dietary guidelines, this was not the case for blue water consumption, suggesting that greater adherence may lead to higher related environmental costs. Furthermore, in this analysis environmental costs outweighed health-related costs. This is in contrast with the FAO’s global estimate that 70 percent of hidden agrifood systems costs are health-related (8). In the Dutch context, these findings suggest that the monetary benefits associated with improved dietary adherence may be larger through reductions in environmental impacts than through health gains. However, these estimates are not directly comparable due to differences in scope (e.g., a national analysis rather than a global assessment, and the limited set of diseases considered in the present analysis), data sources (e.g., reliance on national data versus aggregate databases such as FAOSTAT), and methodological approaches used to quantify and monetise externalities. Overall, our study demonstrates that adhering to dietary guidelines can help reduce hidden costs across multiple domains. By quantifying the hidden costs of suboptimal diets, we provide evidence that improving adherence generates economic gains through both health and environmental pathways. Greater integration of economic and sustainability considerations into dietary recommendations could further enhance these benefits (Discussed in Section 4.2.).

### 4.2 Deriving dietary guidelines for health, sustainability, and the economy

Dietary guidelines are voluntary in nature, which makes them less coercive compared to regulatory or fiscal interventions (65), such as taxation or mandatory restrictions. They also have the potential to shape food systems, for instance, by serving as a basis for product reformulation within the food industry (66). However, their non-compulsory nature and their tendency to be ignored by consumers (65), leads to limited compliance, which reduces their intended impact. This indicates that there is a clear need for improvement in both their design and implementation. Therefore, to maximise their effectiveness in shaping population-level diets, dietary guidelines must be derived to go beyond traditional nutritional adequacy and reduction of disease risk. An agrifood systems approach could also be considered (8), which aims to achieve sustained improvements across multiple interconnected outcomes (e.g., health, economic, and environmental dimension) (67). For instance, explicitly linking dietary recommendations with their long-term societal and economic consequences ensures that diet is viewed not only as an individual health issue but also as a determinant of national welfare, given that economic growth can be facilitated by a healthier population through labour force expansion and productivity gains (68).

Building on this, incorporating sustainability explicitly into dietary guidelines, as advised by the FAO and the World Health Organization (WHO) (69), and especially emphasising the necessary reduction of animal-sourced foods (70), can ensure that such guidelines are better aligned with environmental targets. By including sustainability within dietary recommendations, guidelines not only address nutritional and health objectives, but also contribute to broader societal outcomes. For instance, while promoting sustainable diets that respect environmental boundaries, it can simultaneously help reduce climate change-related effects on health and prevent associated productivity losses (71). How environmental sustainability is included in dietary guidelines is also of importance. According to Sinclair et al. (72), evidence-based dietary guidelines need to justify the recommendations, provide actionable steps, and set quantified targets to enable consumers to adopt sustainable diets which substitute animal-sourced foods for healthier and environmentally friendly alternatives. We also emphasise that the types of food recommended is crucial to highlight. For instance, while the dietary guidelines in the Netherlands promote the consumption of fatty fish (14), it does not specify which species. This omission is critical, because when considering both health effects and environmental impacts, certain high-impact species (e.g., cultivated salmon and eel) are priced below their actual societal impact since their environmental costs offset their health benefits (73). This underscores the need for dietary guidelines to incorporate more specific recommendations that account for both nutritional value and sustainability metrics. Providing such specificity could help consumers make informed choices that optimise their health while minimising environmental harm.

Overall, this integrated approach positions dietary guidelines as a voluntary and preventive tool capable of supporting systematic change across health, economic, and environmental domains. This approach can also be valuable for policy makers and advisors, as it offers a cost-effective mechanism (74), to address multiple societal challenges simultaneously. It is important to acknowledge that information-based policies are often less effective than other market-based policies (75). Furthermore, adherence to such guidelines is influenced by factors including food prices, dietary habits, and sociodemographic characteristics (76). Therefore, it is essential that dietary guidelines must be complemented by supportive measures, such as education campaigns and economic policies (77), to enhance their impact for health improvement and facilitate sustained behavioural change.

### 4.3 Limitations and future research

This study has some limitations that should be acknowledged. First, the DNFCS sample is subject to underreporting of total energy intake (19) and self-reporting biases (e.g., overreporting of healthy foods), which systematically biases diets toward appearing healthier. As such, the estimated burden of poor diets in this study is likely underestimated, and the potential benefits of dietary improvements may be greater than estimated. Second, this study relied on the DHD15 index as the primary measure of dietary quality. While this index reflects adherence to the national dietary guidelines, it does not account for the consumption of explicitly unhealthy foods, such as crisps, confectionery, or other ultra-processed snack items, as these are not explicitly included in the guidelines. Although these product groups are not necessarily associated with diseases in themselves, their nutrient profile, characterised by high levels of fat, sugar and salt, are well-established dietary risk factors. Consequently, by not capturing these products, the index may not fully capture the adverse effects of poor dietary quality, particularly among individuals whose diets include high amounts of such processed foods. Furthermore, the DHD15 is primarily food-based and does fully capture the complexity of nutrient-focused dietary assessment, which may limit its sensitivity in detecting diet-health relationships. Future studies should complement the DHD15 with other indices that capture both healthy and unhealthy components of the diet to provide a more a complete assessment of diet quality. Another limitation concerns the focus on only the 20 percent worst and best dietary adherence, rather than examining the entire population distribution. While this approach provides clear contrasts between extreme dietary patterns and facilitates interpretation, it underestimates the full burden attributable to suboptimal diet across the entire population. Avoided diet-related health costs may already be realised by shifting individuals from the lowest adherence quintile to the second quintile, as modest improvements in dietary quality can reduce exposure to diet-related risk factors and lower the associated health and environmental burden. The same mechanism would apply with any shift from a lower adherence to a higher adherence quintile, and in reality, is not bound to quantile border, but applies for any small shift even within quintiles. Expanding analyses to include intermediate dietary patterns, provided there are sufficiently distinct HR/RR differences across intermediate quintiles to enable quantification, could yield a more complete assessment of diet-related costs and should be explored in future research. This would help to clarify how much health, environment, and economic gain can be achieved through incremental dietary improvements across the population, rather than through shifts between extreme dietary patterns. Furthermore, this study examines the total hidden costs per quintile and does not analyse them by socio-demographic or socio-economic factors. Yet, disparities across income and gender influence who benefits and who benefits these hidden costs (8). For future interventions, such analyses will be necessary to ensure equity in addressing these costs. Regarding the methodological choices, a 36-h workweek was assumed to estimate the productivity losses; however, in 2023 the average working hour was 32.2 (78). Implying that these results represent a maximum estimate rather than the average.

Another uncertainty relates to the use of HRs from Voortman et al. (16). The distribution of dietary intake across quintiles in this study, which were retrieved from the DNFCS, may not fully correspond to those in Voortman’s analysis, which could lead to some misalignment between the exposure levels underlying the HRs and those observed in the population data considered in this study. The scope of diet-related diseases included in this study is also limited. While the selected diseases represent major contributors to diet-related morbidity and mortality, they do not capture the full range of conditions influenced by diet. For instance, type 2 diabetes mellitus (T2DM) caused by poor diets, which accounted for 70.3 percent of new cases globally in 2018 (79), was not included in the morbidity analysis. This exclusion was based on findings from Voortman et al. (16) who did not observe a significant association between better adherence to the Dutch dietary guidelines and reduced risk of T2DM. Although den Braver et al. (80) reported that high adherence compared to low adherence was associated with a lower incidence of T2DM, this association was non-significant after adjustments for Body Mass Index (BMI). Given these mixed findings, T2DM was excluded from the present analysis. The sensitivity analyses (see Supplementary Tables 3, 4) indicated that including T2DM-related costs would increase overall productivity costs and the monetised DALY estimates. However, this does not change the main conclusion of this study. Furthermore, the result of this study suggests that the largest hidden health costs arise from COPD. However, this finding should be interpreted with caution, as limited evidence was found on the relationship between diet and COPD when the 2015 Dutch dietary guidelines were formulated. Overall, the cost estimates presented in this paper should be considered conservative and indicative rather than exhaustive, as they likely underestimate the total burden of diet-related disease.

Lastly, it should be noted that the focus of this study was exclusively on hidden costs. Yet, the costs of food choices should ideally be assessed more broadly, considering not only hidden costs, but also potential societal benefits. Brooks and Diaz-Bonilla (81) argue that hidden costs estimations often exclude countervailing societal benefits, suggesting a more balanced assessment that considers both costs and benefits would better capture the full societal implications of food system changes. For instance, reducing meat consumption may lower healthcare costs and GHG, but at the same time it could affect the incomes and livelihoods of livestock farmers. Similarly, policies promoting healthier diets could shift demand toward plant-based foods, creating new market opportunities while challenging existing agricultural sectors. Incorporating both costs and benefits into such analyses would offer a more comprehensive understanding of these interdependencies and make necessary compromises more transparent, thereby fostering broader acceptance and support for transformative food system policies.

## 5 Conclusion

This study set out to estimate the hidden health and environment costs associated with dietary patterns that deviate from the Dutch dietary guidelines by comparing the population with the poorest adherence to those with the highest adherence. Productivity losses related to diet-related NCDs and DALYs were monetised for the health component. For the environmental component, six environmental impacts were monetised. Our results suggest that if the Dutch adult population with low dietary adherence adopted the dietary patterns observed in the highest adherence group, the combined societal cost savings would include EUR 410 million in avoided productivity losses. When considering the value of DALY, the economic gains would range from EUR 910 million to EUR 1.8 billion. Additionally, the EUR 3.0 billion annual difference in environmental costs between low and high adherence suggests that improved adherence could help prevent environmental costs. By explicitly comparing low and high adherence, our study shows that strengthening dietary guidelines adherence could deliver co-benefits for health, the economy, and the environment. These results reinforce calls for policies and interventions that promote healthier eating patterns as a cost-effective means of reducing the hidden costs embedded in current dietary habits and transforming the agrifood system toward health and sustainability goals.

## Data Availability

The raw data supporting the conclusions of this article will be made available by the authors, without undue reservation.

## Footnotes

1 These are not considered hidden costs in this study as they represent transfers within the healthcare system rather than external costs to society.
