# Supplementary material for: The hidden costs of Dutch dietary choices: quantifying the health and environmental costs attributable to suboptimal diets
Source: Front Nutr. 2026 Mar 23;13:1759095. doi: 10.3389/fnut.2026.1759095 (PMC13051510; doi:10.3389/fnut.2026.1759095)
Supplement: Supplementary file 1 [file Data_Sheet_1.PDF]

Supplementary material

Supplementary table 1. Overview of data sources.

Input data	Year	Source	Details/ additional comments
**Health**			
Food consumption data	2019-2021	Dutch National Food Consumption Survey (DNFCS) (1)	The Dutch National Food Consumption Survey 2019-2021 is freely available upon request from the National Institute for Public Health and the Environment (RIVM). https://www.wateetnederland.nl/publicaties-en-datasets/datasets
Absolute prevalence Chronic Obstructive Pulmonary Disease (COPD), stroke, and colorectal cancer	2023	Vzinfo.nl (2, 3) and Netherlands Cancer Registry (NCR) (4)	
Deaths coronary heart disease (CHD), stroke, type 2 diabetes mellitus (T2DM), colorectal cancer and lung cancer	2023	Vzinfo.nl (5)	
Hazard Ratio (HR)		Voortman et al. (2017) (6)	Quintile 5 (Q1 as reference):<br>All-cause mortality: 0.86 (0.78–0.95)<br>Stroke: 0.74 (0.60–0.91)<br>COPD: 0.72 (0.57–0.87)<br>Colorectal cancer: 0.71 (0.51–0.99)<br><br>These results were based on a confounder model adjusted for cohort, age at dietary assessment, sex, smoking status, educational level, employment status, total energy intake, physical activity, and BMI.
Lost production days due to COPD, stroke, and colorectal cancer	2007-2018	NEMESIS-2 (Netherlands Mental Health Survey and	COPD: 40.6 days<br>Stroke: 30 days<br>Colorectal cancer: 47.6 days

Input data	Year	Source	Details/ additional comments
		Incidence Study-2) (7)	
Labour cost per day	2022	National Health Care Institute (8)<br><br>CAO Rijk (10)	Labour cost per hour (2022): EUR 39.88. Labour cost per hour (2023): EUR 41.41, Consumer Price Index (CPI) considered: 1.04, using data from Statistics Netherlands (9).<br><br>Average working hours per week: 36 hours Average working hour per day: 36 /5 working days = 7.2 hour.
Employment rates	2023	Statistics Netherlands (11)	
Average annual working hours	2023	CAO Rijk (10)	1,822 hours
Discount rate		Guidelines for economic evaluations in healthcare (8)	3%
Disability-adjusted life years (DALYs)	2022	Vzinfo.nl (12)	
Monetisation for DALYs		SEO Amsterdam Economics guidelines for social cost-benefit analyses in the Netherlands (SCBAs) (13)	Range of EUR 50,000 to 100,000 considered.
Healthcare costs related to COPD, stroke, and colorectal cancer	2019	RIVM (14)	Including, hospital care, primary care, medicine and medical aids, ambulance care, and all other healthcare providers, public care and prevention, elderly care, and healthcare management.
**Environment**			
Environmental impact of daily intake	2024	Dutch life cycle assessment	

Input data	Year	Source	Details/ additional comments
		(LCA) Food Database (15, 16)	
Monetisation of environmental impacts	2021	CE Delft (2023) Handbook of Environmental Prices (17)	CPI considered: 1.14, using data from Statistics Netherlands (9).<br><br>CO_2 autonomous annual price increase for from 2021 to 2023: 3.5%<br><br>Changes in actual disposable income compared to the previous year (18):<br>2021 = 1.6%<br>2022 = 0.6%<br>2023 = 1.4%<br><br>Income elasticity (19):<br>Lower: 0.3<br>Central: 0.65<br>Upper: 1.0
Population figures	2023	Statistics Netherlands (20)	

Supplementary table 2. Direct healthcare costs associated with low adherence to the Dutch dietary guideline, by sex and disease.

	**Males**	**Females**	**Total**
	Costs (in million EUR)	Costs (in million EUR)	Costs (in million EUR)
Direct costs[1]			
Colorectal cancer	33	26	59
COPD	19	28	47
Stroke	54	41	95
**Total**			**201**

Sensitivity analysis – Type 2 Diabetes Mellitus (T2DM)

Since the HR for quintile 5 for type 2 diabetes mellitus (T2DM) was not statistically significant and was greater than 1.0 (HR 1.20; 95% CI 0.94-1.55) in Voortman et al. (2017) (6), the lower bound 0.94 was used for the sensitivity analysis. Using the NEMESIS-2 study (7), productivity losses were assumed to amount to 37 days.

Supplementary table 1. Attributable cases and productivity losses associated with low adherence compared to high adherence to the Dutch dietary guidelines, by sex, disease, morbidity, and mortality.

	**Males**		**Females**		**Total**	
	Number of cases attributable	Productivity costs (in million EUR)	Number of cases attributable	Productivity costs (in million EUR)	Number of cases attributable	Productivity costs (in million EUR)
***Morbidity*[1]**						
Colorectal cancer	1357	9	1139	7	2496	16
COPD	13,571	101	13,653	95	27,224	196
Stroke	11,788	60	9,570	46	21,359	106
T2DM	6220	46	4618	32	10,838	78
					**61,917**	**396**
Mortality						
CHD	70	16	24	3	94	19
Colorectal cancer	40	11	28	8	68	19
Lung cancer	101	20	90	14	191	34
Stroke	44	9	32	5	76	14
T2DM	22	5	9	1	31	6
					**460**	**92**
**Total**						**4**88

Note. CHD = Coronary Heart Disease; COPD = Chronic Obstructive Pulmonary Disease; T2DM = type 2 diabetes mellitus.

Supplementary table 2. Attributable disability-adjusted life years (DALYs) and value of life associated with low adherence compared to high adherence to the Dutch dietary guideline, by sex and disease.

	**Males**		**Females**		**Total**	
	Attributable DALY	Value[1] (in million EUR)	Attributable DALY	Value (in million EUR)	Attributable DALY	Value (in million EUR)
Colorectal cancer	2158	108–216	1674	84–167	3832	192–383
COPD	4416	221–442	5062	253–506	9478	474–948
Stroke	2622	131–262	2265	113–226	4887	244–488
T2DM	594	30–59	401	20–40	995	50–99
					**19,192**	**960–1918**

Note. COPD = Chronic Obstructive Pulmonary Disease; [1] = Values are given as a range based on a value of EUR 50,000 to 100,000 per Quality-Adjusted Life Years (QALYs).

Sensitivity analysis – Friction cost approach (FCA)

The productivity losses under the FCA were estimated in accordance with the Dutch guideline for economic evaluations in healthcare (8). Based on the labour cost per day, days of absenteeism per disease, an average working week of 36 hours (7.2 hours per day), the friction cost per person was estimated and then multiplied by the attributable cases.

Supplementary table 5. Attributable cases and productivity losses associated with low adherence compared to high adherence to the Dutch dietary guidelines, by sex, disease, morbidity, and mortality.

	**Males**		**Females**		**Total**	
	Number of cases attributable	Productivity costs (in million EUR)	Number of cases attributable	Productivity costs (in million EUR)	Number of cases attributable	Productivity costs (in million EUR)
Colorectal cancer	1357	7	1139	5	2496	12
COPD	13,571	72	13,653	68	27,224	140
Stroke	11,788	43	9,570	36	21,359	79
					**61,917**	**231**

Note. Chronic Obstructive Pulmonary Disease.

References

1. van Rossum CTM, Sanderman-Nawijn EL, Brants HAM, Dinnissen CS, Jansen-van der Vliet M, Beukers MH, et al. The Diet of the Dutch. Results of the Dutch National Food Consumption Survey 2019-2021 on Food Consumption and Evaluation with Dietary Guidelines. Bilthoven, the Netherlands: National Institute for Public Health and the Environment, (2023) Contract No.: RIVM rapport 2022-0190.
2. Beroerte | Leeftijd En Geslacht | Huisartsencijfers: Vzinfo [updated 17 December 2024; cited 19 June 2025]. Available from: https://www.vzinfo.nl/beroerte/leeftijd-en-geslacht/huisartsencijfers.
3. Copd | Leeftijd En Geslacht | Huisartsencijfers: Vzinfo [updated 18 December 2024; cited 19 June 2025]. Available from: https://www.vzinfo.nl/copd/leeftijd-en-geslacht/huisartsencijfers.
4. Nkr Cijfers: Netherlands Cancer Registry [cited 19 June 2025]. Available from: https://nkr-cijfers.iknl.nl/viewer/prevalentie-per-jaar?language=nl_NL&viewerId=6c1b455d-a0ef-43a3-8da4-9fa48a4d3329.
5. VZinfo. Sterftecijfers: VZinfo.nl [cited 19 June 2025]. Available from: https://www.vzinfo.nl/sterfte/sterftecijfers.
6. Voortman T, Kiefte-De Jong JC, Ikram MA, Stricker BH, Van Rooij FJA, Lahousse L, et al. Adherence to the 2015 Dutch Dietary Guidelines and Risk of Non-Communicable Diseases and Mortality in the Rotterdam Study. *European Journal of Epidemiology* (2017) 32(11):993–1005. doi: 10.1007/s10654-017-0295-2.
7. de Graaf R, Tuithof M, van Dorsselaer S, ten Have M. Verzuim Door Psychische En Somatische Aandoeningen Bij Werkenden: Resultaten Van De ‘Netherlands Mental Health Survey and Incidence Study-2’ (Nemesis-2). Utrecht: Trimbos Instituut, (2011).
8. Hakkaart-van Roijen L, Peeters S, Kanters T. Kostenhandleiding Voor Economische Evaluaties in De Gezondheidszorg: Methodologie En Referentieprijzen. Herziene Versie 2024. Zorginstituut Nederland, (2024).
9. Statistics Netherlands (CBS). Consumentenprijzen; Prijsindex 2015=100: Statistics Netherlands (CBS) [updated 14 January 2025; cited 2025 29 January 2025]. Available from: https://opendata.cbs.nl/#/CBS/nl/dataset/83131NED/table.
10. Uitleg Van Cao-Partijen. Versie Bij Cao Rijk 2022-2024 Met Indexaties Per 1 Juli 2023 En Uitlegteksten Roosteren. CAO Rijk, (2023).
11. Statistics Netherlands (CBS). Arbeidsdeelname; Kerncijfers: Statistics Netherlands (CBS) [cited 19 June 2025]. Available from: https://opendata.cbs.nl/statline/#/CBS/nl/dataset/85264NED/table?dl=9B1CA.
12. VZinfo. Ziektelast in Daly's | Ziektelastcijfers: VZinfo [cited 19 June 2025]. Available from: https://www.vzinfo.nl/ziektelast-in-dalys/ziektelast/interactief-cijferoverzicht.
13. SEO Economisch Onderzoek. Werkwijzer Voor Kosten-Batenanalyse in Het Sociale Domein. SEO Economisch Onderzoek,, (2016).
14. VZinfo. Kosten Van Ziekten: VZinfo [cited 19 June 2025]. Available from: https://www.vzinfo.nl/kosten-van-ziekten.
15. RIVM. Database Milieubelasting Voedingsmiddelen - Database Versie 23 September 2024 Bilthoven: RIVM (2024) [cited 2025 14 January 2025]. Available from: https://www.rivm.nl/voedsel-en-voeding/duurzaam-voedsel/database-milieubelasting-voedingsmiddelen.
16. Vellinga RE, Garcia Valicente M, Sanderman-Nawijn EL, van Bakel M, Hollander A, Temme EHM. De Milieubelasting, Eiwitinname En -Ratio Van De Voedselconsumptie in

Nederland (2019-2021) Bilthoven, the Netherlands: RIVM, (2024) Contract No.: RIVM-briefrapport 2024-0101.
17. de Vries J, de Bruyn S, Boerdijk S, Juijn D, Bijleveld M, van der Giesen C, et al. Environmental Prices Handbook 2024: Eu27 Version - Methodical Justification of Key Indicators Used for the Valuation of Emissions and the Environmental Impact Delft: CE Delft, (2025) April 2025. Report No.
18. Statistics Netherlands (CBS). Kerngegevens Sectoren; Nationale Rekeningen: Statistics Netherlands [updated 14 April 2025; cited 6 May 2025]. Available from: https://www.cbs.nl/nl-nl/cijfers/detail/85881NED#KredietAanPrivateSectorUltimoStand_10.
19. de Bruyn S, de Vries J, Juijn D, Bijleveld M, van der Giesen C, Korteland M, et al. Handboek Milieuprijzen 2023. Methodische Onderbouwing Van Kengetallen Gebruikt Voor Waardering Van Emissies En Milieu-Impacts. Delft: CE Delft, (2023) Contract No.: Publicatienummer: 23.220175.034.
20. Bevolking Op 1 Januari En Gemiddeld; Geslacht, Leeftijd En Regio: Statistics Netherlands [updated 26 May 2025; cited 19 June 2025]. Available from: Bevolking op 1 januari en gemiddeld; geslacht, leeftijd en regio.
